# Interruptions of intensive care nurses during patient care: A potential obstacle to patient safety promotion

**DOI:** 10.1186/2197-425X-3-S1-A716

**Published:** 2015-10-01

**Authors:** C Monteiro, M Pedreira, AFM Avelar, DM Kusahara, MA Peterlini

**Affiliations:** Federal University of São Paulo, Pediatric Nursing, São Paulo, Brazil

## Introduction

Patient safety promotion requires a systemic perspective of healthcare organizations to mitigate and prevent human errors, providing adequate structure; designing processes and creating a safety culture that support the best practice of healthcare professionals. Nurses have a crucial role on patient safety, as they are able to promote an efficient, effective, timely, evidenced-based, and patient centered nursing care.

## Objectives

To verify the occurrence and the sources of interruptions of intensive care nurses during patient care.

## Methods

Observational study of nurses of one pediatric intensive care unit (PICU) and one adult intensive care unit (AICU) from a University Hospital in Brazil. The PICU had eight beds and the AICU had fourteen beds. Observation of nurses' activities was carried out during the day shift comprising 90 hours of data collection. The time spent in patient care, presence and type of interruptions were verified. Variables related to demographic characteristics of nurses, patient and intensive care provided were collected. Data were analyzed through absolute and relative frequencies, mean (+standard deviation), median, Pearson correlation coefficient and Mann-Whitney tests (p≤0.05).

## Results

In both units during the study period 15 nurses were observed, with a median of 29.9 years of age, 93.3% were female, 73.3% had less than five years of work in intensive care, and weekly workload of 44.3(+14.5) hours. During the studied period a total of 457 patients were analyzed with a median of NEMS (Nine Equivalents of Nursing Manpower Use Score) of 30; the nurse to patient ratio was 1:4 in the PICU and 1:2 in the AICU. A total of 1,325 activities were observed and 824 (62.2%) comprised patient care. The other observed activities were related to care management (16.7%), personal activities (10.7%) and unit management (10.4%). Of the 824 activities of patient care 280 (34.0%) were interrupted, with one to six interruptions of the same activity, comprising a total of 476 interruptions analyzed; the number of interruptions per hour was 8.2. The main sources of interruptions were other nurses or nursing technicians (44.1%) and medical staff (17.6%). Total time spent with interruptions during patient care was 267 minutes (7.6%); each nurse spent 4.6 minutes per hour of direct patient care to resolve interruptions. The longer interruptions were caused by lack of supplies. No significant correlation was obtained between NEMS and interruptions (p = 0.083). The years of work in intensive care (< or >5 years) did not influenced interruptions (p = 0.295).

## Conclusions

Nurses were frequently interrupted during patient care, mainly for nursing and medical staff. Such interruptions may compromise attention and patient safety.

